# Associations of anthropometric indices with body adiposity for assessing cardiovascular risk in people living with HIV: a cross-sectional study

**DOI:** 10.7717/peerj.18833

**Published:** 2025-10-20

**Authors:** Elaine Alana Duarte Fernandes, Suerda Teixeira, Phelipe Wilde De Alcântara Varela, Júlio César Medeiros Alves, Paulo Francisco de Almeida-Neto, Carlos Gomes, Breno Guilherme de Araújo Tinôco Cabral, Paulo Moreira Silva Dantas

**Affiliations:** 1 Health Sciences Center, Universidade Federal do Rio Grande do Norte, NATAL, Rio Grande do Norte, Brazil; 2 Nutrition Department, Universidade Federal do Rio Grande do Norte, NATAL, Rio Grande do Norte, Brazil; 3 Physical Education Department, Universidade Federal do Rio Grande do Norte, Natal, Rio Grande do Norte, Brazil

**Keywords:** HIV serodiagnosis, Cardiovascular disease, Body composition, Viral RNA, Nutrition

## Abstract

**Background:**

The presence of HIV infection is associated with an elevated risk of developing cardiovascular disease (CVD), which can be attributed to a range of factors, related to the virus itself. These include increased levels of inflammation and immune activation. Following the introduction of antiretroviral therapy, people living with HIV (PLHIV) began to exhibit an increase in body weight. However, this was also associated with an elevated risk of developing metabolic disorders. The aim of this study was to examine the association between anthropometric indices of cardiovascular risk—waist-to-hip ratio (WHR), waist-to-height ratio (WHtR) and conicity index (CI)—with body composition and time of infection in PLHIV.

**Methods:**

An analytical cross-sectional study consisting of 17 PLHIV of both sexes is conducted. The following anthropometric indices were measured: body weight, height, body mass index (BMI), waist and hip circumference, body fat (BF), WHR, WHtR and CI. For the purposes of statistical analysis, the agreement between the methods used for the diagnosis of cardiovascular risk was verified using the Kappa coefficient. The associations were then verified using the Pearson’s “r” coefficient. The level of significance was set at *p* < 0.05.

**Results:**

The results of the association analyzes between the anthropometric indices WHR, WHtR and CI indicated that WHR and CI were inversely correlated with total BF (kg). Consequently, there was consensus regarding CVD risk between WHR and CI, as well as between the three indices collectively. However, no significant association was observed between the anthropometric indices WHR, WHtR and CI and the duration of HIV infection.

**Conclusion:**

Therefore, it can be concluded that when evaluating the risk of CVD in PLHIV, it is more reliable to associate WHR, WHtR and CI together than to use these indices separately, and total BF can be associated with CI or WHR for a risk assessment.

## Introduction

It has been established that HIV infection causes human immunodeficiency, which is associated with an increased risk of cardiovascular disease (CVD) (Raddusa et al., 2024). This is thought to be due to the effects of chronic immune activation and systemic inflammation (Dirajlal-Fargo & Funderburg, 2022). Despite a reduction in mortality rates among people living with HIV (PLHIV) due to the advent of antiretroviral therapy (ART), these individuals are twice as likely to develop CVD, resulting in a higher mortality rate (Perkins et al., 2023; Shah et al., 2018). The proportion attributable to CVD in PLHIV increased from 0.36% in 1990 to 0.92% in 2015, with a notable rise in disability-adjusted life years (Fragkou et al., 2023; GBD 2017 HIV collaborators, 2019). The mechanisms of CVD include traditional risk factors such as obesity, dyslipidemia, smoking, visceral adiposity and diabetes, as well as non-traditional HIV-related factors such as increased inflammation and immune activation (Grinspoon et al., 2019; Liu et al., 2024). These factors result in an elevated risk of thrombosis and vulnerable atherosclerotic plaques, even in PLHIV with low to moderate traditional risk factors (Hanna et al., 2017).

Although weight gain following the initiation of ART is beneficial for individuals with low body weight, it increases the risk of developing metabolic diseases, including CVD (Bailin et al., 2020). The advent of ART has been accompanied by alterations in adipose tissue, which in turn have given rise to a rise in systemic inflammation due to changes in the profile of immune cells (Koethe, 2017). The additional risk of CVD in PLHIV may be explained by the interaction of HIV-specific factors with excess adiposity (Bailin et al., 2020). Despite the increase in life expectancy, the World Health Organization (WHO) emphasizes the importance of quality of life given that chronic ART is associated with numerous adverse effects, including lipodystrophy (systemic metabolic changes and adipose tissue distribution), which modify body structure and affect self-esteem, thereby reducing adherence to ART (Raddusa et al., 2024). In clinical practice, the risk of CVD is commonly analyzed using anthropometric indices to assess body composition. However, it should be noted that anthropometric indices were developed for subjects without HIV, such as the waist-to-hip ratio and waist-to-height ratio.

It is therefore necessary to investigate the association between the diagnosis of CVD and body fat (BF) in PLHIV, as well as to verify the diagnostic agreement between different anthropometric indices. In this sense, the present study hypothesizes that high BF and prolonged ART are associated with a greater risk of CVD in PLHIV. Therefore, the aim of this study is to analyze the association between anthropometric indices of cardiovascular risk—waist-to-hip ratio (WHR), waist-to-height ratio (WHtR) and conicity index (CI)—with BF and time of infection in PLHIV, with a view to improving the classification and assessment of risk in this population.

## Materials & Methods

### Study design

The present study is quantitative in nature, employing a cross-sectional, analytical and descriptive approach. In order to ensure the quality and transparency of the reporting of our cross-sectional study, we consulted the STROBE (Strengthening the Reporting of Observational Studies in Epidemiology) guideline (Vandenbroucke et al., 2014) and incorporated the items that it recommends into our report.

### Sample and population

The study population comprised 20 individuals with HIV, aged between 28 and 59 years, of both sexes and engaged in regular physical activity (≥ 5 days/week and ≥ 150 minutes/week). Three volunteers were excluded from the study: one due to malnutrition and two for failing to attend all stages of the process. This resulted in a total of 17 participants at the conclusion of the study. The sample size was made for convenience. Data collection took place from March to june 2024, with the procedures carried out over four non-consecutive days with each volunteer, and there was no follow-up period.

The research was conducted at the Human Movement Laboratory (LABMOV) of the Department of Physical Education at the Federal University of Rio Grande do Norte (UFRN). Participants were recruited through conventional means in an extension project aimed at providing physical exercise guidance and nutritional advice to improve the health, quality of life, and well-being of people living with HIV (PLHIV).

### Ethical considerations

The present study was subjected to ethical analysis and received approval from the Research Ethics Committee of the University Hospital Onofre Lopes of the Federal University of Rio Grande do Norte, bearing the approval number 75829023.2.0000.5292. It is noteworthy that all participants were provided with a copy of the free and informed consent form, which they duly signed, thereby indicating their willingness to take part in the procedures of the present study. In doing so, they were fully aware of and agreed to abide by the stipulations set out in the Declaration of Helsinki.

### Study assessments and procedures

Individuals who consented to participate in the study responded to a form containing sociodemographic data and then underwent an anthropometric assessment. The body mass index (BMI), defined as weight in kilograms divided by height in meters squared, was used as the indicator for the anthropometric assessment, with classification in accordance with the World Health Organization (WHO) cutoff points (Bailin et al., 2020). Body mass was determined by means of an electronic scale (MIC-RS232; Micheletti^®^, São Paulo, Brazil) with a capacity of 500 kg, while height was assessed using a portable floor stadiometer (Est-223, Balmak^®^, São Paulo, Brazil) (Lohman, Roche & Martorell, 1988).

To ascertain waist circumference and hip circumference, an inelastic and flexible fiberglass anthropometric tape was employed, with a scale of 0–150 cm and a resolution of 0.1 cm (Sanny^®^, São Paulo, Brazil). The waist circumference was measured with the individual in an upright position, at the midpoint between the last rib and the iliac crest. To measure hip circumference, the examiner positioned themselves adjacent to the subject in order to more accurately visualize the level of maximum gluteal extension. The anthropometric tape was then placed in a horizontal plane, extended over the skin without compressing the soft tissues, in the most prominent region of the hip, between the waist and the thigh (Coelho & Amorim, 2007). This procedure was repeated three times in order to obtain the average circumference value.

Additionally, the WHR and the WHtR were calculated based on anthropometric measurements. The WHtR was calculated by dividing the waist circumference measurement (in centimeters) by the height measurement (in centimeters). For WHtR, the optimal cutoff point of 0.5 balances sensitivity and specificity, indicating that WHtR values greater than or equal to this cutoff are associated with elevated cardiovascular risk (Brazilian Association for the Study of Obesity Metabolic Syndrome, 2016). The WHR was calculated by dividing the waist circumference measurement (in centimeters) by the hip circumference measurement (in centimeters). In order to ascertain the type of central fat distribution according to the WHR, the classification proposed by the World Health Organization (WHO) (1995) was employed. This entailed considering the WHR to be above the recommended level for women when ≥0.85 and for men when ≥0.90.

In order to identify cardiovascular risk, the conicity index (CI) was employed as a parameter, which is determined through measurements of waist circumference (in meters), height (in meters) and weight (in kilograms). The CI was calculated using the mathematical equation proposed by Valdez (1991), which is based on weight, height and waist circumference measurements. The CI was calculated using the following formula: CI = Waist circumference ÷ 0.109 √Body mass ÷ Height. The CI cutoff point was considered to be 1.25 for men and 1.18 for women (Pitanga & Lessa, 2014).

### Body composition

The body composition of the participants was examined using dual-energy X-ray absorptiometry (DXA) (Prodigy Advance model, GE Lunar software (Version 15.0.0); GE^®^, Madison, WI, USA). The data employed in the study were total BF in kilograms (kg), percentage of total BF and android/gynoid ratio (A/G). The assessment of body fat was conducted in accordance with the standards established by Pollock & Wilmore (1993), with the following average percentages considered: males 18–20% (26 to 35 years), 21–23% (36 to 45 years), 24–25% (46 to 65 years old) and females 29–31% (46 to 55 years old) and 30–32% (56 to 65 years old). The DXA equipment was configured as follows for the assessments: The whole-body assessment was conducted with the following settings: voltage (kV) 76.0, current (mA) 0.150, and radiation dose (µGy) 0.4, which is classified as a very low dose with no associated health risks.

### Statistical analysis

The normality of the data was verified using the Shapiro–Wilk test and Q–Q plots. Thus, the data related to the WHR, WHtR and CI indices indicated a non-parametric distribution. However, the other data indicated a parametric distribution. In this sense, to characterize the sample, we presented the data descriptively using average, standard deviation (SD), median, minimum and maximum (continuous data) and frequency distribution (categorical and/or nominal data). For the association analyses, we used the appropriate non-parametric statistics. The agreement of cardiovascular risk diagnosis between the methods was verified using the Kappa coefficient (exact test of kappa and Fleiss’ Kappa), which is interpreted by magnitude (Cohen, 1992): Absence: <0; Bad: 0−0.1; Weak: 0.2−0.3; Moderate: 0.3−0.5; Substantial: 0.6−0.7; Almost complete: ≥0.8. The associations were verified by Spearman’s “Rho” coefficient (r), which was interpreted by magnitude (Cohen, 1992): small (less than 0.20), medium (greater than 0.20 and less than 0.50), and large (greater than 0.50). Kappa and association analyses were performed using the open-source software Jamovi^®^ (version 2.3.18; Jamovi, Sydney, Australia). For all analyses in the present study, *p* < 0.05 was considered statistically significant.

Finally, due to the low sample size (*n* = 17), we performed post-hoc analyses of the sample power for the r coefficient results. To this end, we used the open-source software G*Power^®^ (version 3.1), in the configuration: “T” family tests to verify the power of post-hoc correlation analyses. The sample power was considered adequate when equal to or >0.8 (Cohen, 1988).

## Results

The sociodemographic information pertaining to the participants is presented in Table 1. The majority of participants were male, with an average age of 46 years, single marital status and completed high school. More than half of the volunteers had a monthly income of between 1 and 2 basic salaries and lived in a rented house.

**Table 1:** Sociodemographic characteristics and ART regimen of the study population (*n* = 17).

**Parameters**	**Total** ** (%)**	**Average (SD)**
**Sex**		
Male	14 (82.4)	–
Female	3 (17.6)	
**Age years**	–	46 (9.9)
**Marital status**		
Single	14 (82.4)	–
Married	1 (5.9)	
Divorced	2 (11.8)	
**Education**		
Complete high school	11 (64.7)	–
Complete College	6 (35.3)	
**Income**		
Up to 1 BS	7 (41.2)	–
About 1 and 2 BS	7 (41.2)	
Equal or greater than 3 BS	3 (17.6)	
**Home**		
Rented	11 (64.7)	–
Own home	3 (17.6)	
Ceded	3 (17.6)	
**Infection time**		
< 11 years	12 (70.6)	
>11 years	5 (29.4)	
**ART**		
Lamivudina	14	
Dolutegravir	16	
Tenofovir	4	
Atazanavir	1	
Ritonavir	3	
Biovi	1	
Darunavir	2	

**Notes.**

ART antiretroviral therapy BS Salary base (R$ 1.412,00) SD Standard deviation

Regarding the time of HIV infection, the minimum time reported was 3 years and the maximum was 25 years, with the majority having carried the virus for more than 11 years. The time of infection was extracted into <11 years and >11 years, with 11 years being the median time of infection for our sample.

With regard to BMI, the majority of PLHIV were classified as normal weight (58.8%), although the majority also exhibited a total body fat classification of “very poor” (52.9%). The majority of participants exhibited risk indicators for WHR (76.5%) and WHtR (64.7%) measurements. Moreover, the CI and the A/G assessment were classified as risk for 58.8% and 94.1% of individuals, respectively (Table 2).

**Table 2:** Anthropometric parameters, classification of anthropometric indices of cardiovascular risk, chronic diseases, and CVD history of the sample (*n* = 17).

**Parameters**	**Total (%)**	**Average (SD)**	**Median (Minimum; Maximum)**	**Shapiro–Wilk (*p*-value)**
**Weight** (Kg)	–	69.3 (9.7)	69.3 (55.8; 90.1)	0.15
**BMI**		24.2 (3.0)	25.3 (20.9; 32.3)	0.12
Normal weight	10 (58.8)			
Overweight	6 (35.3)			
Obesity	1 (5.9)			
**Total body fat** (%)		22.3 (9.0)	22.3 (12.6; 45.7)	0.09
Good/average	5 (29.4)			
Fair	2 (11.8)			
Poor	1 (5.9)			
Very poor	9 (52.9)			
**History of CVD**		–	–	–
Yes	1 (5.9)			
No	16 (94.1)			
**Comorbidity related to CVD**		–	–	–
Yes	6 (35.3)			
No	11 (64.7)			
**WHR**		0.9 (0.1)	0.90 (0.65; 1.01)	0.02^*^
With risk	13 (76.5)			
Without risk	4 (23.5)			
**WHtR**		0.5 (0.1)	0.53 (0.39; 0.61)	0.28
With risk	11 (64.7)			
Without risk	6 (35.3)			
**Conicity index**		1.3 (0.1)	1.25 (0.97; 1.38)	0.18
With risk	10 (58.8)			
Without risk	7 (41.2)			
**Rate A/G**		1.3 (0.4)		0.02^*^
With risk	16 (94,1)			
Without risk	1 (5,9)			

**Notes.**

BMI body mass index % percentage SD Standard deviation Kg kilogram CVD cardiovascular disease A/G Android/Gynoid WHR Waist-Hip Ratio WHtR Waist-Height Ratio CI 95% 95% confidence interval of the correlation coefficient r

*p*-value < 0.05.

Regarding comorbidities related to CVD, six of the participants have at least one diagnosis of a disease that may be associated with CVD (hypertension and hypercholesterolemia). However, when analyzing CVD history, only one of the participants reported that they had an acute myocardial infarction.

The concordance of CVD risk between different anthropometric indices was statistically evaluated (Table 3). The WHR and WHtR indices demonstrated no statistically significant agreement, as did the CI and WHtR. However, there was agreement in CVD risk between WHR and CI (*Z* = 2.5; *p* = 0.014). Similarly, when all three indices were considered together, there was also agreement (*Z* = 3.4; *p* = 0.001).

**Table 3:** Association between anthropometric indexes of cardiovascular risk.

**Parameters**	**Risk**	**Exact Kappa**			**Fleiss’ Kappa**		
		**Coeficient**	**Z**	***p* value**	**Coeficient**	**Z**	***p* value**
**WHR with CI**	Yes	0.6	2.5	** 0**.014^*^	–	–	**–**
	No	0.6	2.5	** 0**.014^*^			
**WHR with WHtR**	Yes	0.4	1.8	0.074	–	–	–
	No	0.4	1.8	0.074			
**CI with WHtR**	Yes	0.4	1.6	0.120	–	–	–
	No	0.4	1.6	0.120			
**WHR with WHtR with CI**	Yes	–	–	–	0.5	4.7	< 0.001^*^
	No				0.5	4.7	< 0.001^*^

**Notes.**

WHR Waist-Hip Ratio WHtR Waist-Height Ratio CI Conicity index

* *p*-value < 0.05.

Magnitude: *Absence:  < 0; Bad: 0–0.1; Weak: 0.2–0.3; Moderate: 0.3–0.5; Substantial: 0.6–0.7; Almost complete:*≥*0.8*.

The results of the association analysis (Table 4) indicated that the anthropometric indices WHR and CI, when associated with total BF (kg), were found to be significant. It is noteworthy that the sample size found for such associations was high (>0.8), indicating that the sample size was adequate for these findings.

**Table 4:** Association between anthropometric indices of cardiovascular risk with total body fat and duration of HIV infection.

**Indexes**	**Body fat (Kg)**				**Infection time**			
	**r**	**CI 95%**	**Power**	***p* value**	**r**	**CI 95%**	***p* value**	**Power**
**WHR**	−0.693	−0.700; 0.200	0.958	0.002^*^	−0.061	−0.603; 0.305	0.816	0.06
**Conicity index**	−0.606	−0.669; 0.196	0.835	0.010^*^	0.165	0.027; 0.270	0.528	0.101
**WHtR**	−0.254	−0.370; 0.197	0.173	0.326	0.067	0.035; 0.210	0.798	0.057

**Notes.**

Kg Kilograms WHR Waist-Hip Ratio WHtR Waist-Height Ratio CI 95% 95% confidence interval of the correlation coefficient r

* *p*-value < 0.05.

Magnitude: small (less than 0.20), medium (greater than 0.20 and less than 0.50), and large (greater than 0.50).

However, the anthropometric indices WHR, WHtR and CI did not indicate a significant association with the duration of HIV infection (assessed in years).

## Discussion

The objective of the present study was to investigate the association between the risk of CVD and body composition, as well as the duration of HIV infection. The primary findings indicated that the risk of CVD, as assessed by WHR, WHtR, and CI, was correlated with BF, but not with the duration of HIV infection. Furthermore, there was a significant agreement between WHR and CI in diagnosing the risk of CVD in PLHIV.

The significant results had a moderate to substantial magnitude (0.5–0.6), indicating a relevant and significant effect, which suggests a balance between practical relevance and statistical significance, strengthening the interpretation of the effect as something concrete and applicable, especially in scenarios where extreme variations are not expected or are less desirable (Cohen, 1988).

We highlight that the post-hoc statistical power of the significant associations identified in the present study (WHR with body fat and CI with body fat) was adequate (>0.8), indicating that the sample size was sufficient for these data (Table 4). However, for the non-significant associations (WHtR with body fat and infection time, and WHR and CI with infection time), the post-hoc statistical power was low, suggesting that the sample size may not have been sufficient to detect significant associations in the mentioned terms (Faul et al., 2007).

### Relationship between HIV, body composition and cardiovascular diseases

A comprehensive understanding of health conditions, particularly the presence of complications in PLHIV, enables the identification of high-risk patients and the implementation of targeted preventive measures. Many of these complications have the potential to elevate cardiovascular risk, negatively impacting quality of life and treatment adherence (Miot, 2016). Consequently, there is a clear necessity to ascertain which indices are most effective in classifying the risk of CVD in this population.

In this context, an analysis of body composition must be conducted in conjunction with an evaluation of anthropometric indices. This is because, in the present study, despite the majority of participants having a BMI classified as eutrophic, body adiposity was deemed to be very poor. Consequently, an assessment of BMI alone may result in an inaccurate diagnosis by failing to consider BF. This finding can be attributed to the intrinsic link between HIV and alterations in adipose tissue, which are associated with an increased risk of developing CVD (Grinspoon & Carr, 2005).

The human immunodeficiency virus (HIV) has been observed to act in adipose tissue by triggering immune activation and chronic inflammation. This is achieved through the infiltration of macrophages into the tissue and the secretion of adipokines and cytokines. The indirect effects of the virus result in a reduction in mitochondrial content and mitochondrial dysfunction in adipose tissue (Giralt, Domingo & Villarroya, 2011). Even in the absence of overt lipodystrophy, adipose tissue dysfunction gives rise to lipotoxicity, which presents as dyslipidaemia, hepatic steatosis and insulin resistance. This significantly contributes to the development of systemic metabolic changes observed in PLHIV (Grinspoon & Carr, 2005; Giralt, Domingo & Villarroya, 2011).

Furthermore, the use of antiretroviral drugs in the treatment of HIV affects the distribution of adipose tissue, predisposing to metabolic complications such as type 2 diabetes mellitus, arterial hypertension, endothelial dysfunction and altered production of cytokines and adipokines (Bailin et al., 2020; Giralt, Domingo & Villarroya, 2011; Giralt et al., 2010). These changes are similar to those found in metabolic syndrome (Srinivasa & Grinspoon, 2014), increasing cardiovascular risk in these patients (Bovolini et al., 2021).

A study carried out with elderly without HIV identified BF as the most significant risk factor when associated with CI and WHtR, indicating that the strength of the association between these indices and BF was more specific (Bedimo, 2008). In another study with this same population, BMI and waist circumference were highly correlated with BF%, while WHR demonstrated a weaker relationship with BF%, with differences by sex (Milagres et al., 2019). Both results indicate that the association of these anthropometric indices with BF has a specific correlation, even not in a public with HIV.

Considering PLHIV, a study evaluated the agreement between waist circumference, WHR, and BMI as proxies for cardiometabolic risk, finding a strong linear association and correlation between these indices even after adjusting for gender and ART status. Waist circumference and WHR minimally agreed with BMI in identifying HIV patients at increased cardiometabolic risk (Andreacchi et al., 2021). Other research pointed to indicators of central abdominal fat as the most useful for estimating longitudinal changes in body fat in HIV individuals, with initial WHR being the best predictor of this indicator at the end of the follow-up period (Dimala et al., 2018). These findings reinforce that WHR is relevant to assess the risk of CVD, reinforcing the results of the present study.

Adipose tissue, which is susceptible to the detrimental effects of HIV infection, is also affected by a range of antiretroviral medications (Linares et al., 2020). In contrast to the period preceding the advent of ART, when PLHIV experienced widespread loss of weight, lean mass and body fat, ART is currently associated with patients who are of normal weight or overweight, who maintain muscle mass but present significant alterations in the distribution of adipose tissue, leading to the development of HIV lipodystrophy syndrome (SLHIV) (Bedimo, 2008; Beraldo et al., 2017; Carr, 2003).

Although the full mechanisms of SLHIV are not fully understood, the prevailing hypothesis suggests that metabolic and body composition changes represent the primary mechanism. This understanding enabled us to establish a correlation between outcomes and the most commonly prescribed antiretroviral drugs. Among antiretrovirals, protease inhibitors (PIs) and nucleoside and nucleotide analog reverse transcriptase inhibitors (NRTIs) are the classes most closely associated with alterations in the function and biology of adipose tissue (Giralt, Domingo & Villarroya, 2011; Giralt et al., 2010; Beraldo et al., 2017).

PIs have been linked to adverse effects on adipocytes, including the inhibition of differentiation and the negative regulation of transcription factors associated with adipogenesis. Additionally, they have been shown to induce apoptosis in adipocytes and contribute to insulin resistance (Carr, 2003). Recent adipose tissue biopsy studies in PLHIV have demonstrated an alteration in the expression of peroxisome proliferator-activated receptor gamma (PPARγ), which is the main regulator of adipocyte differentiation (Grunfeld et al., 2008). Conversely, NRTIs have been linked to the inhibition of mitochondrial RNA transcription, mitochondrial DNA depletion and organelle dysfunction, through the inhibition of DNA polymerase γ. This process is thought to be a significant contributor to the development of SLHIV (Finkelstein et al., 2015). It is noteworthy that individuals with HIV who are on ART tend to exhibit an increase in total BF in the trunk and a reduction in the percentage of BF in the limbs compared to individuals without HIV, even in the absence of clinically apparent HIV. These changes persist even after ART is changed (Vidal et al., 2012; Grunfeld et al., 2010).

Among the study participants, six were identified with lipodystrophy (Santos et al., 2019) and were using ART with the NRTI lamivudine, which suggests a potential correlation between the administration of this medication and the onset of lipodystrophy. Furthermore, four of these individuals had an overweight BMI and five had a poor or very poor BF percentage.

It is important to note that, in contrast to the general population, individuals with HIV are at a significantly elevated risk of developing CVD, which tend to manifest at an earlier age (*i.e.,* below 45 years) (Schouten et al., 2014; Shah et al., 2018). Over the past decade, there has been a notable increase in efforts to prevent CVD in PLHIV. This has led to a significant focus on the development of new cardiovascular risk prediction techniques, which are crucial for clinical decision-making and early intervention. These techniques can provide valuable insights into the level of risk, facilitating more informed clinical management and enabling the identification of individuals who may benefit from early intervention (D’Agostino Sr, 2012; Achhra et al., 2022).

### Practical applicability

The present study was conducted at an opportune time, as it verified the agreement between different anthropometric indices for diagnosing the risk of CVD and analyzed the association between the results of different anthropometric indices. This allows for reflection on the findings, enabling the identification of the anthropometric index or combination of indices that offer the most accurate prediction of CVD in PLHIV. This approach allows for a more precise clinical assessment of this group. This suggests that, in a more targeted clinical approach, the combination of the WHR, WHtR and CI indices will provide greater precision in the assessment of cardiovascular risk than the use of each index alone.

The Longitudinal Study of Adult Health (ELSA-Brazil) investigated the relationship between obesity-related measures based on body characteristics and CVD in a large sample of women and men. The evaluation of different combinations revealed that WHR in conjunction with BF was the most predictive factor for men, while WHR in combination with BMI proved to be a more determinant factor for women. Furthermore, it was observed that the presence of increased BF or BMI, in conjunction with a high waist circumference or WHR, significantly increased the 10-year risk of developing CVD. In conclusion, the results highlight that combinations involving at least one indicator of general obesity and one indicator of central obesity were the main predictors of cardiovascular risk over 10 years, emphasizing the importance of integrated approaches to the analysis of CVD profiles (Eickemberg et al., 2019; Silva et al., 2020; Almeida et al., 2020; Almeida, Matos & Aquino, 2021).

The data presented herewith support clinical practice with less technology or financial resources available, since not all professionals have at their disposal a tool classified as high standard for assessing body composition, such as DXA, which facilitates the assessment of body composition and enables associations with other PLHIV health data. Therefore, the assessment of the risk of developing CVD in PLHIV can be carried out using these associated anthropometric indices, preferably all three (WHR, WHtR and CI).

### Limitations and suggestions for future studies

A limitation of this study is the relatively small sample size, which is a common challenge in research involving people living with HIV. Despite the small sample size, the power of the sample was considered to be strong. However, we suggest that in future studies, multicenter surveys be carried out to explore the different characteristics of the sample from different locations, with a greater chance of achieving a high sample size.

As a suggestion for future studies, we suggest a longitudinal follow-up, which would give a greater ability to assess causality.

It would be beneficial to conduct further laboratory tests, particularly in relation to lipid profile standards (total cholesterol, HDL-cholesterol, LDL-cholesterol, non-HDL-cholesterol, VLDL-cholesterol and triglycerides), apolipoproteins, C-reactive protein (CRP), and other relevant biochemical markers. This would facilitate the investigation of potential associations between biochemical and anthropometric patterns in subsequent studies.

## Conclusions

It can be concluded that there is a significant association between total body fat, the conicity index, and waist-hip ratio. This demonstrates that an assessment of cardiovascular risk in people living with HIV becomes more reliable when these two anthropometric indices are associated with body fat. Conversely, the time of infection with the virus did not demonstrate significance among the anthropometric indices. With regard to the assessment of cardiovascular disease risk, an agreement was observed between the waist-hip ratio and the conicity index. Furthermore, there was agreement when both indices were considered simultaneously, indicating that their combined use may be more effective when evaluating individuals with HIV who are on antiretroviral therapy.

##  Supplemental Information

10.7717/peerj.18833/supp-1 Supplemental Information 1 Raw data

10.7717/peerj.18833/supp-2 Supplemental Information 2 STROBE checklist

## Additional Information and Declarations

### Data Availability

The following information was supplied regarding data availability: The raw data is available in the Supplementary File.
