# Supplementary material for: Associations of anthropometric indices with body adiposity for assessing cardiovascular risk in people living with HIV: a cross-sectional study
Source: PeerJ. 2025 Oct 20;13:e18833. doi: 10.7717/peerj.18833 (PMC12548633; doi:10.7717/peerj.18833)
Supplement: Supplemental Information 2 [file peerj-13-18833-s002.docx]

STROBE Statement—Checklist of items that should be included in reports of ***cross-sectional studies***

|  | **Item No** | **Recommendation** |
| --- | --- | --- |
| **Title and abstract** | 1 | (*a*) Indicate the study’s design with a commonly used term in the title or the abstract Page 1 |
|  |  | (*b*) Provide in the abstract an informative and balanced summary of what was done and what was found - Page 1-2 |
| **Introduction** | | |
| Background/rationale | 2 | Explain the scientific background and rationale for the investigation being reported - Page 2 |
| Objectives | 3 | State specific objectives, including any prespecified hypotheses - Page 2 |
| **Methods** | | |
| Study design | 4 | Present key elements of study design early in the paper - Page 3 |
| Setting | 5 | Describe the setting, locations, and relevant dates, including periods of recruitment, exposure, follow-up, and data collection - Page 3 |
| Participants | 6 | (*a*) Give the eligibility criteria, and the sources and methods of selection of participants - Page 3 |
| Variables | 7 | Clearly define all outcomes, exposures, predictors, potential confounders, and effect modifiers. Give diagnostic criteria, if applicable - Page 3-4 |
| Data sources/ measurement | 8* | For each variable of interest, give sources of data and details of methods of assessment (measurement). Describe comparability of assessment methods if there is more than one group - Page 3-4 |
| Bias | 9 | Describe any efforts to address potential sources of bias - Page 3 |
| Study size | 10 | Explain how the study size was arrived at - Page 3 |
| Quantitative variables | 11 | Explain how quantitative variables were handled in the analyses. If applicable, describe which groupings were chosen and why - Page 4-5 |
| Statistical methods | 12 | (*a*) Describe all statistical methods, including those used to control for confounding - Page 4-5 |
|  |  | (*b*) Describe any methods used to examine subgroups and interactions - Page 4-5 |
|  |  | (*c*) Explain how missing data were addressed - Page 4-5 |
|  |  | (*d*) If applicable, describe analytical methods taking account of sampling strategy - Page 4-5 |
|  |  | (*e*) Describe any sensitivity analyses - Page 4-5 |
| **Results** | | |
| Participants | 13* | (a) Report numbers of individuals at each stage of study—eg numbers potentially eligible, examined for eligibility, confirmed eligible, included in the study, completing follow-up, and analysed - Page 5, Table 1 |
|  |  | (b) Give reasons for non-participation at each stage - Page 5 |
|  |  | (c) Consider use of a flow diagram - Disregarded |
| Descriptive data | 14* | (a) Give characteristics of study participants (eg demographic, clinical, social) and information on exposures and potential confounders - Page 5, Table 1 |
|  |  | (b) Indicate number of participants with missing data for each variable of interest - Disregarded |
| Outcome data | 15* | Report numbers of outcome events or summary measures - Page 5 |
| Main results | 16 | (*a*) Give unadjusted estimates and, if applicable, confounder-adjusted estimates and their precision (eg, 95% confidence interval). Make clear which confounders were adjusted for and why they were included - Page 5 |
|  |  | (*b*) Report category boundaries when continuous variables were categorized - Disregarded |
|  |  | (*c*) If relevant, consider translating estimates of relative risk into absolute risk for a meaningful time period - Disregarded |
| Other analyses | 17 | Report other analyses done—eg analyses of subgroups and interactions, and sensitivity analyses - Disregarded |
| **Discussion** | | |
| Key results | 18 | Summarise key results with reference to study objectives - Page 5-8 |
| Limitations | 19 | Discuss limitations of the study, taking into account sources of potential bias or imprecision. Discuss both direction and magnitude of any potential bias - Page 5-8 |
| Interpretation | 20 | Give a cautious overall interpretation of results considering objectives, limitations, multiplicity of analyses, results from similar studies, and other relevant evidence - Page 5-8 |
| Generalisability | 21 | Discuss the generalisability (external validity) of the study results - Page 5-8 |
| **Other information** | | |
| Funding | 22 | Give the source of funding and the role of the funders for the present study and, if applicable, for the original study on which the present article is baseds - Page 9 |

*Give information separately for exposed and unexposed groups.
